# ASP-assisted symbolic regression for interpretable modelling of 3D laminar channel flow

**DOI:** 10.1038/s41598-026-49762-y

**Published:** 2026-04-30

**Authors:** Theofanis Aravanis, Grigorios Chrimatopoulos, Mohammad Ferdows, Michalis Xenos, Efstratios Em. Tzirtzilakis

**Affiliations:** 1 Department of Digital Systems, University of the Peloponnese, 231 00 Sparta, Greece; 2 Department of Mechanical Engineering, University of the Peloponnese, 263 34 Patras, Greece; 3 Department of Applied Mathematics, University of Dhaka, Dhaka, 1000 Bangladesh; 4 Department of Mathematics, University of Ioannina, 451 10 Ioannina, Greece; 5 Department of Civil Engineering, University of the Peloponnese, 263 34 Patras, Greece

**Keywords:** Symbolic regression, Answer set programming, Knowledge representation, Fluid mechanics, Interpretability, Data-driven, Symbolic artificial intelligence, Engineering, Mathematics and computing, Physics

## Abstract

Symbolic Regression (SR) offers an interpretable alternative to conventional Machine-Learning (ML) approaches, which are often criticized as “black boxes”. In contrast to standard regression models that require a prescribed functional form, SR constructs expressions from a user-defined set of mathematical primitives, enabling the automated discovery of compact formulas that fit the data and reveal underlying physical relationships. In fluid mechanics, where understanding the underlying physics is as crucial as predictive accuracy, this study applies SR to model three-dimensional (3D) laminar flow in a rectangular channel, focusing on the axial velocity and pressure fields. Compact symbolic equations were derived from numerical simulation data, accurately reproducing the expected parabolic velocity profile and linear pressure drop, and showing excellent agreement with analytical solutions from the literature. To address the limitation that purely data-driven SR models may overlook domain-specific constraints, an innovative hybrid framework that integrates SR with Answer Set Programming (ASP) is also introduced. This integration combines the generative power of SR with the declarative reasoning capabilities of ASP, ensuring that derived equations remain both statistically accurate and physically plausible. The proposed SR/ASP methodology demonstrates the potential of combining data-driven and knowledge-representation approaches to enhance interpretability, reliability, and alignment with physical principles in fluid dynamics and related domains.

## Introduction

Fluid mechanics is essential to many fields, from engineering and environmental science to medical research. However, the complexity of fluid behaviour, especially in turbulent or multiphase systems, makes it challenging to model and understand. Traditional methods, including Computational Fluid Dynamics (CFD), have been highly effective and physically transparent, yet often incur substantial computational cost. On the other hand, modern Machine-Learning (ML) approaches range from interpretable methods to more opaque models such as Artificial Neural Networks. Although ML surrogates are often used in fluid mechanics primarily for computational efficiency, many commonly adopted models still behave as “black boxes”, offering limited physical interpretability—an important limitation in applications where understanding the underlying flow physics is as important as prediction quality.

Against this background, research increasingly turns to data-driven methods for new solutions, and approaches that combine accuracy with clear, understandable models. *Symbolic Regression* (SR) stands out in this space. Unlike typical ML models that operate as “black boxes”, SR identifies mathematical expressions that describe relationships in the data. Rather than relying on a fixed predefined functional form, SR searches over combinations of user-specified mathematical primitives, enabling it to reveal or confirm physically meaningful relationships in fluid mechanics^1^. This feature is invaluable for researchers and engineers who need models that are not just predictive, but also provide insight into the underlying physics.

Despite the interpretability of SR and its successful use in a plethora of domains^2–6^, the application of evolutionary algorithm-based SR in fluid mechanics remains relatively limited. In what follows, we briefly review representative studies that apply SR within fluid-mechanics contexts.

Schmidt and Lipson’s seminal study showed that symbolic methods can automatically distill free-form natural laws (including Hamiltonians/Lagrangians) directly from experimental time series by targeting non-trivial invariants, foreshadowing later physics-aware SR workflows^4^. Praks and Brkić^7^ illustrated SR’s potential by approximating the Colebrook equation for turbulent friction factors, refining initial models through fixed-point iterations to enhance accuracy with low computational cost. Extending SR to particle-fluid interactions, El Hasadi and Padding^8^ proposed new drag correlations for ellipsoidal and spherocylindrical particles, while Milošević *et al.*^9^ unified laminar and turbulent friction models via data-driven symbolic formulations. Sofos *et al.*^10^ applied SR to define a Lennard-Jones fluid descriptor, and Roland *et al.*^11^ used it to model viscous dissipation in polymer extrusion, reducing reliance on costly simulations. For Lennard-Jones fluids, Angelis *et al.*^12^ developed closed-form expressions for viscosity and thermal conductivity spanning dilute to dense regimes, while Alam *et al.*^13^ predicted self-diffusion with interpretable and accurate SR models. In turbulence, Wu and Zhang^14^ enhanced the shear-stress-transport (SST) turbulence model with SR-derived correction terms that generalized across 3D flows, and Chakrabarty and Yakovenko^15^ improved Reynolds-averaged Navier-Stokes (RANS) closures through compact symbolic stress approximations.

Parallel to evolutionary SR, sparse-regression approaches such as SINDy formalize model discovery as selecting a parsimonious set of terms from rich function libraries, enabling scalability to fluid systems and robustness to noise^16^; the PySINDy package operationalizes these ideas with practical tooling and constraint options^17^. In this vein, Reinbold *et al.*^18^ demonstrated the effectiveness of physics-aware sparse regression in experimental settings by reconstructing external force fields from noisy, high-dimensional measurements, illustrating how library-based SR can integrate prior physics to yield interpretable and reliable models. Complementarily, dimensionless-learning frameworks enforce dimensional invariance to recover compact, scale-free variables and their associated scaling relations —along with dimensionally homogeneous differential equations— from scarce, noisy experiments, providing a principled route to interpretable, scale-aware models^19^.

In this article, we investigate the use of SR as an interpretable, data-driven tool for modelling a fundamental laminar fluid-flow problem. Our approach establishes a novel and robust benchmark for interpretable ML models in viscous fluid motion within confined geometries. Instead of prescribing a specific functional form, our SR framework successfully derives explicit expressions for the (axial) *velocity* and *pressure* fields in *three-dimensional* (3D) channels as functions of *spatial coordinates* and the *Reynolds number*. As demonstrated, the derived symbolic expressions closely match the corresponding numerical solutions, reproducing the expected physical behaviour and aligning with established analytical trends. These results suggest that the SR-based expressions offer an interpretable and computationally efficient representation of the underlying flow physics, while remaining consistent with high-fidelity numerical predictions.

**Fig. 1:**
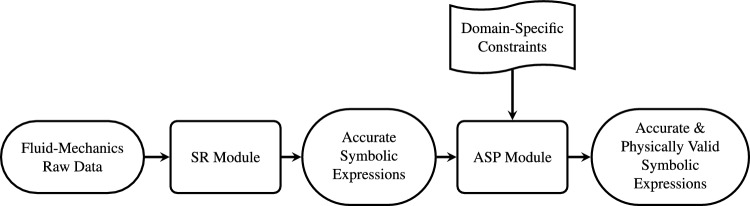
Integrated SR and ASP work-flow. SR generates candidate accurate symbolic expressions from fluid-mechanics raw data. Then, ASP applies domain-specific constraints to filter and select expressions that are both accurate and physically valid.

While, as we show, SR excels in its generative capabilities, it operates within a *purely data-driven* manner, potentially overlooking intricate domain-specific constraints and logical relationships inherent to physical systems. This limitation can lead to the selection of models that, despite their statistical accuracy, *may violate* fundamental physical laws or, in general, domain-specific constraints. To address these challenges, we propose an exploratory integration of SR with the *knowledge-representation* framework of *Answer Set Programming* (ASP)^20–22^. ASP constitutes a declarative-programming paradigm that allows the specification of complex problems and constraints in a high-level (symbolic) language. By blending data-driven and symbolic Artificial Intelligence (AI), the resulting conceptual hybrid SR/ASP framework ensures that the SR-generated symbolic expressions are not only statistically accurate, but also physically plausible, adhering to domain-specific constraints and principles, encoded into ASP (see Fig. 1). This integration offers a promising direction for building versatile and trustworthy AI systems, capitalizing on the complementary strengths of learning from data and reasoning with knowledge^23^. It is worth emphasizing that, while ASP has been extensively applied across a wide range of domains^24,25^, the integration of ASP with SR represents, to the best of our knowledge, the first effort to combine these two powerful frameworks.

On the whole, the present study highlights two major contributions: First, the ability of SR to translate intricate flow dynamics into simple, interpretable equations that effectively balance precision and clarity; and second, the significant role of knowledge-representation techniques in improving the reliability and domain-specific validity of data-driven SR models. These developments open new avenues for incorporating hybrid (data-driven and symbolic) methodologies into efficient computational systems, particularly in high-stakes fluid-dynamics scenarios where the integration of detailed, explainable simulations with real-time, data-driven insights is crucial.

The remainder of the article is organized as follows: The subsequent section (section Mathematical formulation of the fluid-flow problem) sets out the mathematical formulation of the fluid-flow problems under investigation. Thereafter, section The symbolic-regression models introduces the architecture and parameters of the developed SR models. Section Results and discussion is dedicated to the presentation and discussion of the results of the SR models. Section Symbolic Regression Enhanced with Answer Set Programming presents the alluded hybrid SR/ASP approach. The article closes with a concluding section that summarizes the overall contribution and discusses interesting avenues for future research.

## Mathematical formulation of the fluid-flow problem

The governing equations describing (time-independent) fluid flow in three spatial dimensions, without external forces, are the Navier-Stokes equations,

1 $$\begin{aligned} \begin{array}{l} \rho \left( u'\frac{\partial u'}{\partial x'}+v'\frac{\partial u'}{\partial y'}+w'\frac{\partial u'}{\partial z'}\right) = -\frac{\partial p'}{\partial x'} + \mu \left( \frac{\partial ^2 u'}{\partial {x'}^2}+\frac{\partial ^2 u'}{\partial {y'}^2}+\frac{\partial ^2 u'}{\partial {z'}^2}\right) ,\\ \rho \left( u'\frac{\partial v'}{\partial x'}+v'\frac{\partial v'}{\partial y'}+w'\frac{\partial v'}{\partial z'}\right) = -\frac{\partial p'}{\partial y'} + \mu \left( \frac{\partial ^2 v'}{\partial {x'}^2}+\frac{\partial ^2 v'}{\partial {y'}^2}+\frac{\partial ^2 v'}{\partial {z'}^2}\right) ,\\ \rho \left( u'\frac{\partial w'}{\partial x'}+v'\frac{\partial w'}{\partial y'}+w'\frac{\partial w'}{\partial z'}\right) = -\frac{\partial p'}{\partial z'} + \mu \left( \frac{\partial ^2 w'}{\partial {x'}^2}+\frac{\partial ^2 w'}{\partial {y'}^2}+\frac{\partial ^2 w'}{\partial {z'}^2}\right) , \end{array} \end{aligned}$$

complemented by the conservation of mass equation, $\frac{\partial u'}{\partial x'}+\frac{\partial v'}{\partial y'}+\frac{\partial w'}{\partial z'}=0$, where $u',v',w'$ are the dimensional velocity components and $p'$ is the dimensional fluid pressure. Additionally, $\rho$ is the fluid density and $\mu$ is the dynamic fluid viscosity^26^. To derive the non-dimensional form of the governing equations, the following non-dimensional parameters are introduced:

2 $$\begin{aligned} x = \frac{x'}{h}, \quad y = \frac{y'}{h}, \quad z = \frac{z'}{h}, \quad u = \frac{u'\,h\,\rho }{\mu }, \quad v = \frac{v'\,h\,\rho }{\mu }, \quad w = \frac{w'\,h\,\rho }{\mu }, \quad p = \frac{p'\,h^2\,\rho }{\mu ^2}, \end{aligned}$$

where *h* represents the characteristic height^27^.

By substituting the parameters defined in Equation (2) into the dimensional Navier-Stokes equations (Equation (1)), the governing equations are recast in non-dimensional form. Applying the chain rule, in conjunction with the conservation of mass equation, yields the following closed-form expressions for the non-dimensional governing equations:

3 $$\begin{aligned} \begin{array}{l} \frac{\partial \left( u^2\right) }{\partial x} + \frac{\partial \left( uv\right) }{\partial y} + \frac{\partial \left( uw\right) }{\partial z} = -\frac{\partial p}{\partial x} + \frac{\partial ^2 u}{\partial x^2} + \frac{\partial ^2 u}{\partial y^2} + \frac{\partial ^2 u}{\partial z^2},\\ \frac{\partial \left( vu\right) }{\partial x} + \frac{\partial \left( v^2\right) }{\partial y} + \frac{\partial \left( vw\right) }{\partial z} = -\frac{\partial p}{\partial y} + \frac{\partial ^2 v}{\partial x^2} + \frac{\partial ^2 v}{\partial y^2} + \frac{\partial ^2 v}{\partial z^2},\\ \frac{\partial \left( wu\right) }{\partial x} + \frac{\partial \left( wv\right) }{\partial y} + \frac{\partial \left( w^2\right) }{\partial z} = -\frac{\partial p}{\partial z} + \frac{\partial ^2 w}{\partial x^2} + \frac{\partial ^2 w}{\partial y^2} + \frac{\partial ^2 w}{\partial z^2}. \end{array} \end{aligned}$$

It should be noted that applying the non-dimensional parameters of Equation (2) leads to non-dimensional equations that do not explicitly include non-dimensional groups, such as the Reynolds number $\textrm{Re}$. In the present setting, the flow is instead driven by a prescribed constant streamwise pressure gradient *c*, which plays the role of the primary control parameter. A corresponding Reynolds number will be introduced later in this section as a convenient, equivalent parametrization of the operating condition.

The Finite Volume Method (FVM) is employed to discretize Equations (3) and the conservation of mass equation. The FVM discretizes the computational domain into smaller, finite control volumes, called cells^28^. By integrating the governing equations over these control volumes, the system of non-linear partial differential equations is transformed into a larger system of non-linear *algebraic* equations, subject to corresponding boundary conditions. This transformation allows the equations to be solved using methods for non-linear systems, such as Newton’s method. Additionally, the FVM evaluates equations at each cell centroid, enabling the implementation of various boundary conditions^29^.

The algebraic system obtained by the FVM discretization can be written in the form

4 $$\begin{aligned} \begin{aligned} \frac{1}{2}\Delta y \Delta z \left( u_{E}^{2} - u_{W}^{2}\right) +\frac{1}{2}\Delta x\Delta z\left( u_{N}v_{N}-u_{S}v_{S}\right) +\frac{1}{2}\Delta x\Delta y\left( u_{T}w_{T}-u_{B}w_{B}\right) +\Delta y\Delta z\left( p_{E}-p_{P}\right) \\ -\frac{\Delta y\Delta z}{\Delta x}\left( u_{E}-2u_{P}+u_{W}\right) -\frac{\Delta x\Delta z}{\Delta y}\left( u_{N}-2u_{P}+u_{S}\right) -\frac{\Delta x\Delta y}{\Delta z}\left( u_{T}-2u_{P}+u_{B}\right) =0, \\ \frac{1}{2}\Delta y\Delta z\left( u_{E}v_{E}-u_{W}v_{W}\right) +\frac{1}{2}\Delta x\Delta z\left( v_{N}^2-v_{S}^{2}\right) +\frac{1}{2}\Delta x\Delta y\left( v_{T}w_{T}-v_{B}w_{B}\right) +\Delta x\Delta z\left( p_{N}-p_{P}\right) \\ -\frac{\Delta y\Delta z}{\Delta x}\left( v_{E}-2v_{P}+v_{W}\right) -\frac{\Delta x\Delta z}{\Delta y}\left( v_{N}-2v_{P}+v_{S}\right) -\frac{\Delta x\Delta y}{\Delta z}\left( v_{T}-2v_{P}+v_{B}\right) =0, \\ \frac{1}{2}\Delta y\Delta z\left( u_{E}w_{E}-u_{W}w_{W}\right) +\frac{1}{2}\Delta x\Delta z\left( v_{N}w_{N}-v_{S}w_{S}\right) +\frac{1}{2}\Delta x\Delta y\left( w_{T}^{2}-w_{B}^{2}\right) +\Delta x\Delta y\left( p_{T}-p_{P}\right) \\ -\frac{\Delta y\Delta z}{\Delta x}\left( w_{E}-2w_{P}+w_{W}\right) -\frac{\Delta x\Delta z}{\Delta y}\left( w_{N}-2w_{P}+w_{S}\right) -\frac{\Delta x\Delta y}{\Delta z}\left( w_{T}-2w_{P}+w_{B}\right) =0. \end{aligned} \end{aligned}$$

Here, $\Delta x$, $\Delta y$, and $\Delta z$ denote the grid spacing in each coordinate direction, respectively. The velocity components $u_i$, $v_i$, $w_i$, and the pressure $p_{i}$ are evaluated at the control-volume faces $i \in \big \{E,W,N,S,T,B\big \}$ and at the cell center $P$. Additional details regarding the grid structure and control-volume arrangement can be found in the works^30–32^.

The numerical solution of the system of Equation (4) is obtained using an in-house code implemented in MATLAB. The resulting algebraic system is solved on a collocated grid, using Newton’s method combined with trust-region techniques. The solver settings include a structured grid of $50 \times 25 \times 25$ control volumes, resulting in $4 \times 50 \times 25 \times 25$ degrees of freedom (four unknowns $u,v,w,p$ per control volume). Convergence is declared when the Euclidean norm of the residual vector falls below $10^{-7}$.

By assuming that the velocity components $v$ and $w$ are identically zero throughout the flow, Equations (3) simplify significantly. In particular, one obtains $\partial p / \partial x = c$, implying a *linear* pressure drop along the channel length, and

$$\frac{\partial ^2 u}{\partial y^2} + \frac{\partial ^2 u}{\partial z^2} = c,$$

which yields a velocity profile $\tilde{u}$ that depends on the imposed pressure gradient $c$. This profile $\tilde{u}$ is numerically evaluated for various values of $c$, and is subsequently used as the inlet axial-velocity distribution.

The discrete system of Equation (4) is complemented by the following boundary conditions:

5 $$\begin{aligned} \begin{aligned} \text {Inlet:}\quad u = \tilde{u},\quad v = 0,\quad w = 0,\quad \frac{\partial p}{\partial x} = c, \\ \text {Outlet:}\quad \frac{\partial u}{\partial x} = 0,\quad \frac{\partial v}{\partial x} = 0,\quad \frac{\partial w}{\partial x} = 0,\quad p = 0, \\ \text {Walls:}\quad u = 0,\quad v = 0,\quad w = 0,\quad \frac{\partial p}{\partial y} = 0,\quad \frac{\partial p}{\partial z} = 0. \end{aligned} \end{aligned}$$

These conditions imply that the axial velocity component $u$ follows the inlet profile $\tilde{u}$, while the transverse components $v$ and $w$ are identically zero at the inlet. A constant streamwise pressure gradient $c$ is imposed at the channel inlet. At the outlet, homogeneous Neumann boundary conditions are applied to all velocity components, whereas the pressure is fixed to zero. Along the channel walls, no-slip boundary conditions are enforced for the velocity components, and homogeneous Neumann boundary conditions are used for the pressure.

**Fig. 2:**
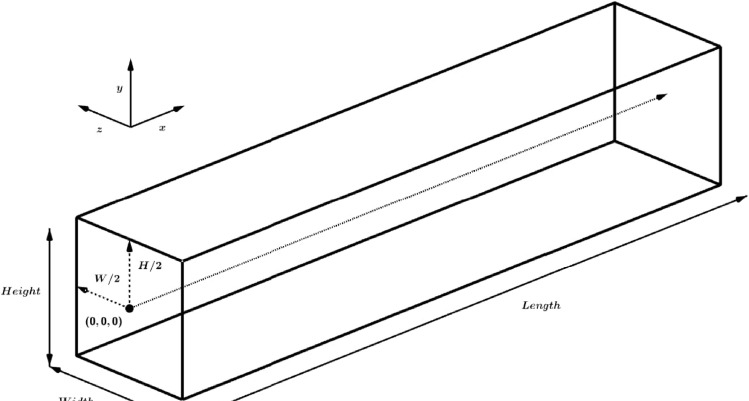
Geometry of the three-dimensional symmetrical rectangular duct. The computational domain spans $(0,L)\times (-H/2,H/2)\times (-W/2,W/2)$. The duct height *H* is used as the characteristic length scale.

In the present study, the fluid-mechanics problem under consideration is the fluid flow in a three-dimensional, symmetrical rectangular duct, with dimensions $\left( 0,L\right) \times \left( -H/2,H/2\right) \times \left( -W/2,W/2\right)$. A visual representation of the geometry of the problem is shown in Fig. 2. Note that the characteristic length *h* appearing in the identities of Equation (2) is assumed to be the height of the duct *H*; i.e., $h=H$. It is also noteworthy that, although the geometry of the problem appears relatively simple, deriving a corresponding analytical solution can be challenging. For example, Kakaç *et al.*^33^, Chapter 3 present an analytical solution expressed as an infinite series, underscoring the complexity of the problem, while also providing approximations that reduce computational costs.

To address the studied problem, numerical data were generated to train and test the Symbolic-Regression models by varying the driving pressure gradient. Specifically, a constant streamwise pressure gradient ranging from $c=-1000$ to $c=-8000$, with a step size of $-1000$, was applied to the Poisson equation $\partial ^2 u/\partial y^2 + \partial ^2 u/\partial z^2 = c$, resulting in distinct fully developed inlet velocity profiles. The duct dimensions are specified as $L = 5$ and $H = W = 1$, and the fluid properties are kept fixed throughout.

As previously mentioned, the non-dimensional parameters of Equation (2) yield governing equations that do not explicitly include the Reynolds number. In this work, the flow regime is therefore most naturally indexed by the imposed pressure gradient *c*. For convenience of interpretation, we additionally compute an effective Reynolds number $\textrm{Re}$ *a posteriori* from the numerical solution *u* via

$$\textrm{Re} = \int _{-W/2}^{+W/2} \int _{-H/2}^{+H/2} u \, dy \, dz,$$

following Tzirtzilakis and colleagues^27^. For a fixed geometry and fluid, the laminar regime considered here ensures a one-to-one, monotonic relationship between the imposed pressure gradient *c*, the characteristic velocity, and the corresponding Reynolds number. Thus, $\textrm{Re}$ functions as a *case-level control parameter*, equivalent to having prescribed $\textrm{Re}$ directly for each simulation. It is therefore not a per-sample function of the target field, but a global descriptor of the flow regime that labels each simulation (i.e., each value of *c*).

The resulting *training dataset* includes the velocity components *u*, *v*, *w*, and the pressure *p* within the 3D channel for operating conditions characterized by $\textrm{Re} \in \big \{34, 105, 174, 209, 279\big \}$, while the *testing dataset* comprises the same quantities for $\textrm{Re} \in \big \{70, 139, 244\big \}$. Consequently, the SR models are evaluated under *interpolation* scenarios with respect to this scalar control parameter. It is noted that the forthcoming analysis excludes the velocity components *v* and *w*, as their magnitudes remain negligible and approach zero throughout the channel, as indicated by the numerical solutions.

## The symbolic-regression models

Symbolic Regression (SR) is a powerful computational technique used to uncover interpretable mathematical relationships that govern data dynamics. Unlike traditional regression methods that rely on a fixed, predefined functional form, SR explores a flexible search space constructed from user-specified mathematical primitives to derive models that are both accurate and interpretable. This flexibility makes SR particularly suited for domains where the underlying physical relationships are unknown or complex.

SR is typically implemented using evolutionary algorithms, which draw inspiration from biological processes like natural selection and genetic mutation^34^. These algorithms treat mathematical expressions as individuals within a population, and evolve them over successive generations to optimize their performance against a fitness criterion, such as minimizing prediction error. The process begins with the generation of an initial population of random mathematical expressions, composed of variables, constants, and operators. Each of these expressions is then evaluated for fitness, based on how accurately it approximates the observed data using predefined metrics like Mean Squared Error or custom loss functions. The population is subsequently evolved by introducing variation through operations such as mutation, which randomly alters parts of an expression, and crossover, which combines segments from two parent expressions to produce new candidates. Finally, a selection and refinement step retains the most accurate and interpretable expressions, gradually improving the population across generations. This biologically inspired work-flow enables SR to efficiently explore a vast space of candidate mathematical relationships, striking a balance between predictive accuracy and interpretability. The outcome is a set of concise, human-readable equations that capture the underlying dynamics of the system being modelled.

For implementing SR in the present study, the PySR tool (version 0.19.4) was employed. PySR is a powerful SR library written in Python and Julia, leveraging techniques such as regularized evolution, simulated annealing, and gradient-free optimization^35^. In the remainder of this section, we detail the design and implementation of the SR models, highlighting their configuration and providing the rationale behind each methodological choice.

**Table 1:** Key parameters of the PySR tool used for the development of the two SR models.

Parameter	Value	Description
niterations	1000	Number of training iterations
binary_operators	+ , − , $*$ , /	Core arithmetic operations
unary_operators	square(x) = x² , cube(x) = x³	Low-order polynomials as building blocks
maxsize	25	Limits expression complexity
nested_constraints	True	Restricts nested square and/or cube operators
variable_names	*X* , *Y* , *Z* , $\textrm{Re}$	Names of input variables *x* , *y* , *z* , $\textrm{Re}$

To begin with, two distinct SR models were developed: One specifically designed to predict the axial velocity *u*, and the other to model the pressure *p* within the 3D channel. In constructing these models, interpretability was encouraged by restricting the maximum allowed expression complexity, and by selecting a domain-motivated set of primitive mathematical operators (e.g., arithmetic operations and low-order polynomial functions). These primitive operators also constrain the search space, thereby preserving computational tractability.

More specifically, the models were trained for 1000 iterations. This setting was supported by trial runs using markedly larger iteration budgets, which indicated that the resulting expressions and error metrics remained effectively unchanged. Core arithmetic operations, such as addition, subtraction, multiplication, and division, formed the basis for constructing expressions. To capture more complex relationships, non-linear operators, including square and cube functions, were also incorporated as “building blocks”. This choice is motivated by fluid-mechanics principles, which indicate that laminar fluid-flow solutions frequently exhibit polynomial-like behaviour — allowing velocity and pressure profiles to be well-approximated by low-order polynomials, as will be further discussed in Subsection Literature’s analytical equations, where we examine relevant *analytical* equations from the literature. It should be stressed that restricting the operator set in a domain-informed manner is standard practice in SR. For example, in the seminal PySR paper, Cranmer notes that “empirical relations are frequently comprised of operators which are unique to one particular field of science, so the equation search strategy must allow for custom operators”^35^, p. 3, underscoring the importance of incorporating domain-specific, user-defined operators within PySR.

To enhance the simplicity and utility of the resulting equations, a number of constraints were introduced. The *complexity* of expressions —expressed by the total count of mathematical operations and terms within each equation— was limited by setting a maximum size (25), ensuring they remained interpretable. Additionally, nested constraints were applied to prevent unnecessary combinations of higher-order functions, such as squaring a squared term. This helped reduce redundancy and promoted concise mathematical representations. Reproducibility was ensured by explicitly configuring PySR in deterministic mode, using a fixed $\texttt {random}\_\texttt {state}$ and disabling parallelism so that repeated runs yield identical results.

As outlined in section Mathematical formulation of the fluid-flow problem, the training and testing datasets comprise the spatial coordinates (*x*, *y*, *z*) together with the Reynolds number ($\textrm{Re}$) as input variables, while the corresponding outputs are the axial velocity (*u*) and the pressure (*p*). Here, $\textrm{Re}$ plays the role of a scalar control parameter that indexes the operating condition of each simulation (through the imposed pressure gradient), since all samples originating from the same numerical run share a *single* $\textrm{Re}$ value. The SR models therefore learn a *parametric* mapping of the form $u = u(x,y,z;\textrm{Re})$ and $p = p(x,y,z;\textrm{Re})$ across different flow regimes, rather than a self-referential dependence of *u* on its own pointwise values. For clarity, the upper-case variables *X*, *Y*, *Z* appearing in the forthcoming SR equations denote the same non-dimensional coordinates as *x*, *y*, *z* used in the mathematical formulation of the fluid-flow problem. A summary of the key parameters and settings employed by the PySR tool is provided in Table 1.

During the training process, the SR models iteratively refine their mathematical expressions, striving to minimize the prediction error quantified by a *loss function* (fitness criterion). PySR uses Mean Squared Error (MSE) as its default loss function to evaluate the performance of candidate equations, adhering to predefined configurations, as outlined in Table 1. For a target quantity $y$, the MSE is defined as $\text {MSE} = \frac{1}{n} \displaystyle \sum _{i=1}^{n} \left( y_i - \hat{y}_i \right) ^2$, where $n$ is the number of data points, $y_i$ represents the reference (actual) value of the $i$-th data point, and $\hat{y}_i$ denotes the SR-predicted value for the $i$-th data point.

Upon completing the training process, PySR produces a collection of candidate models that form the *Pareto front*^35^. In multi-objective optimization, the Pareto front is the set of all Pareto-efficient solutions, namely, the set of solutions that cannot be improved in any one objective without degrading at least one of the other objectives^36^. In our context, the Pareto front represents the optimal trade-off between simplicity (model complexity) and accuracy (error metrics such as MSE or MAE). Formally, a model belongs to the Pareto front if and only if no other model achieves *both* a lower complexity and a lower error. Hence, models on the Pareto front are characterized by their *best balance* between interpretability and predictive accuracy, ensuring robustness and minimizing the risk of overfitting.

## Results and discussion

Having discussed the architecture of our SR models, we turn to the presentation of the derived results concerning the axial velocity *u* and the pressure *p* of the channel.

The equations derived from the SR models are assessed using three primary error metrics: Mean Squared Error (MSE), Mean Absolute Error (MAE), and Normalized Mean Absolute Error (NMAE). The MSE, introduced in Section The symbolic-regression models, serves as the default loss function (fitness criterion) employed by PySR to evaluate candidate expressions. Meanwhile, focusing on the prediction of a target quantity $y$, MAE and NMAE are defined as $\text {MAE} = \frac{1}{n} \displaystyle \sum _{i=1}^{n} |y_i - \hat{y}_i|$ and $\text {NMAE} = \frac{\text {MAE}}{\max (y)-\min (y)}\, 100\%$, where $n$ is the total number of data points, $y_i$ is the reference (actual) value of the *i*-th data point, $\hat{y}_i$ is the SR-predicted value for the *i*-th data point, and $\max (y)$, $\min (y)$ are the maximum and minimum reference values of *y*, respectively.

### Symbolic equations

Against this background, two symbolic equations derived from the implemented SR models are presented below. Selected automatically from the Pareto front of their respective models using the built-in PySR model-selection criterion (model_selection=”best”), these expressions reflect a balance between predictive accuracy and expression complexity rather than minimum loss alone, thereby constituting robust and interpretable candidates for further exploration and analysis. The computation time required for an SR model to generate the full set of Pareto-front equations is approximately 15 minutes on a system equipped with a 13th-generation Intel^®^ Core™ i7–13700 processor (2.10 GHz) and $32$ GB of RAM.

6 $$\begin{aligned} u^{\text {symb}}&= \textrm{Re}\, \Big ( 2.18 - 8.46\, Y^2 \Big )\Big ( 1 - 3.89\, Z^2 \Big ) \end{aligned}$$

7 $$\begin{aligned} p^{\text {symb}}&= \textrm{Re}\, \Big ( 143.43 - 28.69\, X \Big ) \end{aligned}$$

Equation (6) defines the axial velocity $u$ within the channel as a function of the Reynolds number $\textrm{Re}$ and the spatial variables $Y$ and $Z$. The expression incorporates quadratic dependencies on both $Y$ and $Z$, with coefficients that capture the influence of $\textrm{Re}$ on the velocity distribution. The quadratic terms establish a *parabolic* velocity profile, consistent with the expected flow characteristics in such a configuration. The pressure field $p$ is represented symbolically in Equation (7) as a function of the Reynolds number $\textrm{Re}$ and the spatial variable $X$. This equation reveals a negative linear relationship between $p$ and $X$, where the coefficients are scaled by $\textrm{Re}$. The negative correlation between $p$ and $X$ highlights a linear pressure drop across the channel, a common feature in laminar flow systems.

**Table 2:** Measures of accuracy for Equations (6) and (7) of the SR models.

Equation	Complexity	Training Dataset (62.5%)			Testing Dataset (37.5%)		
		MSE	MAE	NMAE	MSE	MAE	NMAE
$u^{\textrm{symb}}$ (6)	17	44.98	4.81	$0.008 \%$	47.62	5.36	$0.010 \%$
$p^{\textrm{symb}}$ (7)	9	$1.68\cdot 10^{-6}$	0.0011	$2.75\cdot 10^{-8} \%$	$1.8 \cdot 10^{-6}$	0.0012	$3.44\cdot 10^{-8} \%$

Table 2 provides a detailed comparison of the symbolic expressions, highlighting both their complexity and performance on the training and testing datasets. The performance is evaluated using the error metrics MSE, MAE, and NMAE, quantifying the accuracy of the symbolic models for the axial velocity ($u^{\textrm{symb}}$) and the pressure field ($p^{\textrm{symb}}$), with results reported separately for the training and testing datasets. As already stated, the complexity of each equation, shown in the second column of Table 2, reflects the total count of mathematical operations and terms within each expression. A higher complexity value suggests a more intricate equation, which often results from the need to capture detailed relationships in the data.

For $u^{\textrm{symb}}$ (Equation (6)), with a complexity of 17, the model achieves a low NMAE of $0.008 \%$ on the training dataset and $0.010 \%$ on the testing dataset. These results demonstrate a highly accurate fit, with slightly better performance observed during training. The pressure field, $p^{\textrm{symb}}$ (Equation (7)), has a notably lower complexity of 9 but achieves remarkable precision. The MSE values for both the training and testing datasets are in the order of $10^{-6}$, while the NMAE is consistently in the range of $10^{-8} \%$. This demonstrates that the symbolic model for $p$ not only captures the underlying trend but does so with minimal error, even for unseen data.

Overall, the results highlight the balance between complexity and accuracy achieved by the SR approach. The models effectively generalize from the training data to the testing data, with minimal loss in accuracy, showcasing their robustness and suitability for modelling fluid velocity and pressure within the channel. The consistent performance across the training and testing datasets suggests that overfitting was effectively mitigated, reinforcing the reliability of the approach.

### Representative plots

**Fig. 3:**
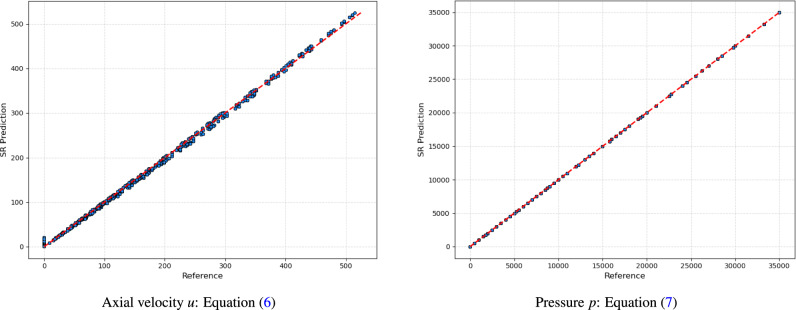
Identity plots for Equations (6) and (7) of the SR models. The red $45^\circ$ line represents a perfect match between the symbolic equation derived from an SR model and the numerical results.

The low error metrics presented in Table 2 are further substantiated by the exceptional alignment observed in the fitted *identity* plots for Equations (6) and (7), which compare the predictions of the SR models against the reference numerical results, as illustrated in Fig. 3.

**Fig. 4:**
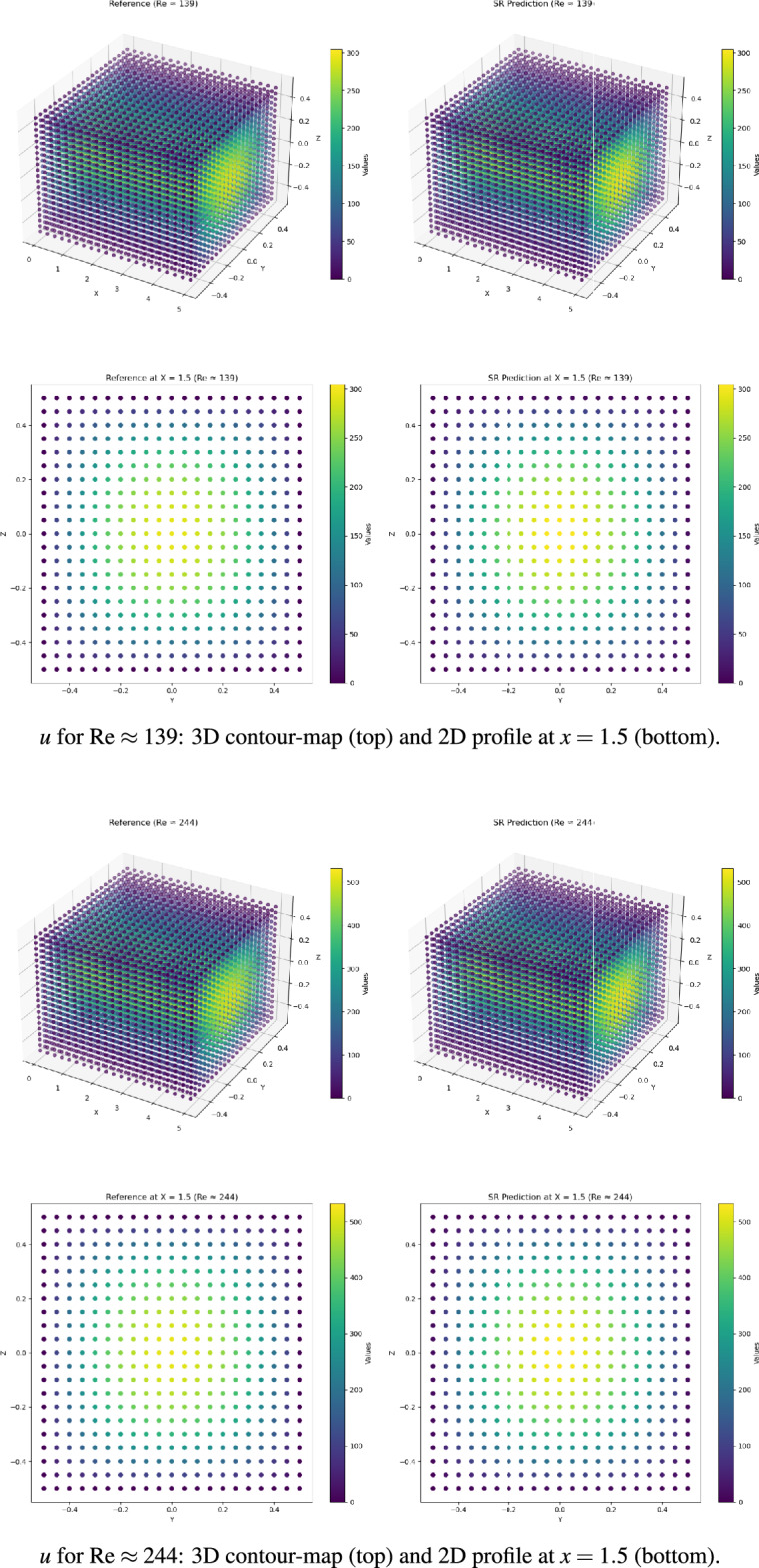
3D contour-maps of the axial velocity *u* within the channel, alongside its 2D profile at a representative vertical cross-section of the channel, for indicative Reynolds numbers of the testing dataset. Depicted are the predicted quantities as generated by the SR model, and the reference quantities as generated by the numerical solutions.

**Fig. 5:**
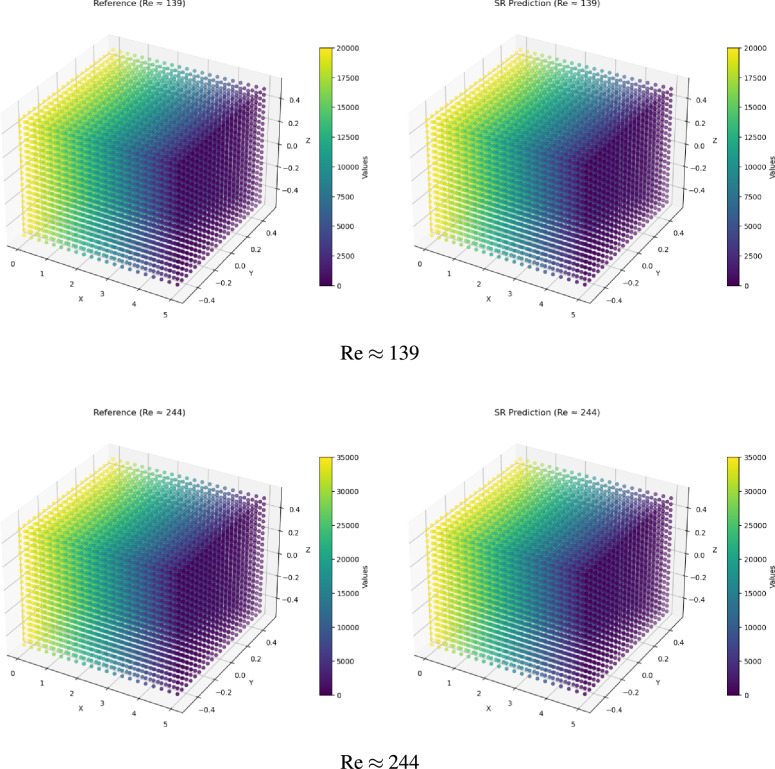
3D contour-maps of the pressure *p* within the channel, for indicative Reynolds numbers of the testing dataset. Depicted are the predicted quantities as generated by the SR model, and the reference quantities as generated by the numerical solutions.

Furthermore, Fig. 4 depicts the 3D contour-maps of the axial velocity *u* within the channel, alongside its 2D profile at a representative vertical cross-section of the channel, for indicative Reynolds numbers of the testing dataset. Depicted are the predicted quantities as generated by the SR model, and the reference quantities as generated by the numerical solutions. Along similar lines, Fig. 5 depicts the 3D contour-maps of the pressure *p* within the channel, for indicative Reynolds numbers of the testing dataset. Depicted are the predicted quantities as generated by the SR model, and the reference quantities as generated by the numerical solutions.

Evidently, the SR models demonstrate exceptional accuracy and interpretability, not only in capturing the flow’s axial velocity field, but also in representing its pressure distribution. For the axial velocity *u*, the derived equation produces nearly indistinguishable results from the reference numerical solution across the tested Reynolds numbers. The predicted flow fields preserve the quintessential parabolic profile, smoothly peaking at the channel’s centerline and tapering off near the boundary walls in accordance with the no-slip boundary conditions. This fidelity to fundamental physical behaviour is quantified by exceedingly low NMAEs, consistently at the order of a few thousandths of a percent. Such negligible discrepancies underscore the model’s remarkable capability to generalize beyond the training dataset and its promise for reducing reliance on high-fidelity, computationally expensive simulations. The SR model developed for the pressure provides an equally compelling representation of the pressure field. The retrieved symbolic expression accurately captures the expected linear pressure gradient along the channel’s longitudinal direction, a hallmark feature of laminar, fully developed flow. The minimal residual errors in pressure predictions, as reflected by the near-vanishing error metrics, attest to the robustness of the model and its ability to encode fundamental fluid-dynamic principles in a compact functional form.

### Literature’s analytical equations

Following the presentation of the concrete results obtained from the SR models, we now delve into *analytical* equations from the literature relevant to laminar fluid flows in 3D channels. A foundational example is the exit velocity profile of an incompressible fluid flowing steadily through a rectangular duct, as illustrated in Fig. 2 of Sect. Mathematical formulation of the fluid-flow problem. This profile is described approximately by the equation

8 $$\begin{aligned} u = u_{\text {max}} \left( 1 - 4\,\frac{y^2}{H^2} \right) \left( 1 - 4\,\frac{z^2}{W^2} \right) , \end{aligned}$$

where $u$ is the axial velocity at any point within the cross-section, $u_{\text {max}}$ is the maximum velocity at the center of the duct, $H$ is the height of the duct in the $y$-direction, $W$ is the width of the duct in the $z$-direction, and $y$, $z$ are the coordinates in the cross-sectional plane^37^, p. 198, Problem P3.18. (For exact solutions of fully developed velocity profiles in flows through rectangular ducts, the reader is referred to the work of Kakaç *et al.*^33^.) In the context of our study, where the duct dimensions are defined as $H = W = 1$ (section Mathematical formulation of the fluid-flow problem), Equation (8) simplifies to

9 $$\begin{aligned} u = u_{\text {max}} \Big ( 1 - 4\, y^2 \Big ) \Big ( 1 - 4\, z^2 \Big ). \end{aligned}$$

The above expression highlights the *parabolic* velocity profile commonly observed in laminar flows through ducts, with $u_{\text {max}}$ at the center and the velocity decreasing symmetrically toward the walls, due to the no-slip boundary conditions.

Building on this foundation, the conducted numerical simulation suggests a direct relationship between the Reynolds number $\textrm{Re}$ and the maximum axial velocity at the centerline of the channel $u_{\text {max}}$, given by

10 $$\begin{aligned} \textrm{Re} \approx \frac{u_{\text {max}}}{2.109}. \end{aligned}$$

By combining Equations (6) and (10), we obtain the following symbolic representation for the velocity profile:

11 $$\begin{aligned} \begin{aligned} u^{\text {symb}}&\approx \frac{u_{\text {max}}}{2.109} \Big ( 2.18 - 8.46\, Y^2 \Big ) \Big ( 1 - 3.89\, Z^2 \Big ) = u_{\text {max}} \Big ( 1.03 - 4.01\, Y^2 \Big ) \Big ( 1 - 3.89\, Z^2 \Big ). \end{aligned} \end{aligned}$$

Evidently, the forms of Equations (9) and (11) are strikingly similar. The symbolic representation in Equation (11), derived from the corresponding SR model, provides an excellent approximation to the analytical solution from the literature, namely Equation (9). The small remaining discrepancy may be due both to the approximate nature of Equation (9) and to the finite spatial resolution of the numerical dataset used to generate the SR training data. As the present problem is steady, temporal discretization does not arise, and the relevant numerical-resolution issue is therefore the spatial one. Quantifying its effect more precisely would require a systematic grid-refinement study, which is left for future work. Similarly, the symbolic representation of the pressure field in Equation (7), derived from the corresponding SR model, aligns closely with the general form of pressure distributions reported in the literature, according to which the pressure *drops linearly* across the length of the channel^37,38^. This close agreement underscores the ability of the developed SR models to capture the fundamental characteristics of the velocity and pressure fields with high fidelity, reinforcing their potential as powerful tools for both theoretical studies and real-world engineering applications.

### Robustness analysis

Although our primary analysis relies on the deterministic dataset generated by the numerical simulation, in this subsection we additionally assess the robustness of the proposed approach by injecting controlled levels of synthetic *noise* into the existing data and retraining the SR models. To this end, we perturbed the target fields by applying independent pointwise random perturbations, while leaving all input variables unchanged. Specifically, each target value was multiplied by a random factor chosen uniformly within $\pm \, 2\%$ of its original value, thereby producing noisy versions of the fields with controlled relative perturbations.

Accordingly, two symbolic equations derived from the Pareto front of the noise-augmented SR models are presented below.

12 $$u^{\mathrm{symb\_noise}} = \mathrm{Re} \left( 2.17 - 8.43 Y^2 \right) \left( 1 - 3.87 Z^2 \right)$$

13 $$p^{\mathrm{symb\_noise}} = \mathrm{Re} \left( 143.42 - 28.68 X \right)$$

Both Equations (12) and (13) remain very close to their noise-free counterparts (Equations (6) and (7), respectively), demonstrating strong robustness to perturbations in the data. This is further confirmed by the corresponding MSE, MAE, and NMAE values on both the training and testing datasets, which exhibit only minor deviations from the deterministic case (cf. Table 2).

It should be stated that the presence of noise generally makes SR model selection more challenging, as it perturbs the fitness values of competing expressions and may broaden the Pareto front. In the present case, however, the effect of the imposed $2\%$ noise remained limited, as the SR procedure recovered the same qualitative forms as in the noise-free setting, with only minor coefficient changes. This supports the robustness of the proposed methodology in both predictive accuracy and equation discovery.

Lastly, we verified that the overall accuracy and stability of the SR models were preserved when the SR operator set was expanded to include trigonometric and exponential functions (cf. Table 1). Even within these richer search spaces, the SR procedure consistently converged to polynomial-type expressions, indicating that the discovered relationships are not an artefact of a restricted operator set, but are consistent with the underlying flow physics.

## Symbolic Regression Enhanced with Answer Set Programming

As already made clear, SR has gained prominence as a robust methodology for discovering mathematical relationships within complex datasets, without presupposing a specific model structure. While SR excels in its generative capabilities, it often operates within a *purely data-driven* paradigm, potentially overlooking intricate domain-specific constraints and logical relationships inherent to physical systems. This limitation can lead to the selection of models that, despite their statistical accuracy, *may violate* fundamental physical laws or lack interpretability within the context of established scientific theories. To address these challenges, we propose an integration of SR with Answer Set Programming (ASP) — a form of *declarative* programming oriented towards difficult combinatorial search problems^20–22^. This innovative *hybrid* approach seamlessly merges data-driven and symbolic Artificial Intelligence into a unified framework, combining the generative power of SR with the declarative reasoning strengths of ASP, which not only enhances model selection, but also ensures adherence to physical principles (refer to Fig. 1 of Introduction). It is important to highlight that, although both ASP and SR have been extensively studied independently, our proposal represents, to the best of our knowledge, the first attempt to integrate these two powerful frameworks.

### Answer set programming

ASP constitutes a *knowledge-representation* paradigm that allows the specification of complex problems and constraints in a high-level, declarative language. Unlike imperative approaches, ASP focuses on describing *what* the solution should satisfy rather than *how* to discover it^20–22^. ASP operates by defining a logic program, namely, *rules* and *facts* that describe the problem domain, allowing an ASP solver to compute *stable models*, also known as *answer sets* — i.e., consistent sets of literals (atomic formulae or their negation) that satisfy all given constraints. This capability makes ASP particularly suitable for tasks that require intricate constraint satisfaction, logical inference, and combinatorial optimization^24,25^.

### Methodology

The integration of ASP with SR involves a systematic work-flow that leverages the strengths of both methodologies. Initially, SR tools like PySR are employed to explore the space of possible mathematical models that fit the fluid-mechanics data. This process yields a diverse set of candidate equations, each characterized by attributes such as complexity, loss (an error metric), and functional form. However, the raw output from SR often includes models that may not comply with domain-specific constraints essential for physical validity.

To imbue the SR-generated models with domain-specific intelligence, the next step involves preprocessing and feature extraction. This can be accomplished using scripting languages like Python, where the symbolic forms of the candidate equations are parsed to extract relevant features. Features of interest may include the presence of specific terms (e.g., $X^4$), functional properties (such as monotonicity or asymptotic behaviour), and dimensional attributes ensuring dimensional consistency. These extracted features are then represented as facts in an ASP-compatible format, effectively translating the symbolic information into a form that ASP can process.

Subsequently, an ASP program can be developed to encapsulate the desired constraints and domain-specific knowledge. These constraints can range from simple exclusions or inclusion of certain terms to more complex rules enforcing physical laws such as conservation of mass and momentum. For instance, the ASP program can include rules that prevent the selection of equations containing non-physical terms (e.g., $X^4$), enforce the selection of equations containing essential parameters like the Reynolds number ($\textrm{Re}$), or ensure that selected models adhere to dimensional homogeneity. Additionally, ASP’s optimization capabilities can be harnessed to balance multiple objectives, such as minimizing both model complexity and prediction error while ensuring compliance with physical constraints.

An ASP solver, such as Clasp, is then employed to process (the propositional form of) the ASP program, alongside the preprocessed facts derived from the SR output. Clasp is a widely used, off-the-shelf ASP solver that incorporates state-of-the-art techniques from Boolean constraint solving^39^. The solver evaluates the rules and constraints, identifying *stable models* that represent valid selections of equations adhering to all specified criteria. These stable models effectively filter out non-compliant equations, retaining only those that are both data-accurate and physically meaningful. The (physically) validated models can then be utilized for further analysis, simulations, or as components of more comprehensive fluid-mechanics theories.

### A concrete example

To demonstrate the practical integration of the proposed SR/ASP hybrid approach, we present a concrete example based on the equations derived by the SR model tailored to the axial velocity *u* of the flow (see section Results and discussion). Recall that these equations are all part of the *Pareto front* of the SR procedure, meaning that each one retains an optimal balance of accuracy and simplicity. The presented example illustrates how SR-generated symbolic expressions can be effectively filtered and validated using ASP to ensure their physical plausibility and adherence to domain-specific constraints. As will be shown, the methodology is highly versatile, allowing for the incorporation of numerous constraints or adaptation to other domains.

First, Table 3 reports all the (14) equations of the Pareto front, identified by the alluded SR model. Each equation varies in complexity and loss (MSE), representing different levels of fit and intricacy in capturing the underlying physical phenomena. Note that the equation corresponding to ${\textbf {ID 9}}$ is Equation (6) of section Results and discussion.

**Table 3:** The equations of the Pareto front, identified by the SR model tailored to the axial velocity *u* of the flow. Observe that the complexity of equations remains up to 25, as constrained by the corresponding limitation outlined in Table 1 of section The symbolic-regression models.

ID	Complexity	Loss	SR Equation for the Axial Velocity *u*
0	1	37396.16	*X*
1	2	31757.25	$X^3$
2	3	13316.42	$0.91 \ \textrm{Re}$
3	5	13316.14	$0.91 \ \textrm{Re} - 0.63$
4	6	10670.45	$\textrm{Re} - 427.88\ Y^2$
5	8	9246.47	$\textrm{Re} - 849.34\ Y^2 + 76.16$
6	10	7431.94	$1.41\ \textrm{Re} - 5.62\ \textrm{Re}\ Z^2$
7	13	7417.14	$1.41\ \textrm{Re} - 5.62\ \textrm{Re} \ Z^2 - 1.41 \ Y^2$
8	15	3620.02	$(1.70 - 7.35\ Y^2)\, (\textrm{Re} - 400.53 \, Z^2) + 11.5$
${\textbf {9}}$	${\textbf {17}}$	**44.98**	$\textbf{Re} \left( \mathbf {2.18} - \mathbf {8.46}\ \mathbf {Y^2} \right) \left( \textbf{1} - \mathbf {3.89}\ \mathbf {Z^2} \right)$
10	20	25.31	$(2.15 -8.45 \ Y^2 + Z^2)\, (\textrm{Re} -3.98 \ \textrm{Re} \ Z^2) + 0.35$
11	22	25.07	$(2.15 - 8.45 \ Y^2 + Z^2)\, (\textrm{Re} -3.98 \ \textrm{Re} \ Z^2 + 0.53) - 0.41$
12	24	25.02	$(2.15 - 8.45 \ Y^2 + Z^2) \ (\textrm{Re} -3.98 \ \textrm{Re} \ Z^2 + Y^2) - Y^3$
13	25	22.96	$(2.15 - 8.45 \ Y^2 + Z^2) \, (\textrm{Re} -3.98 \ \textrm{Re} \ Z^2 + 8.45 \ Y^2) - Y$

While the symbolic expressions of Table 3 achieve an optimal balance between accuracy and simplicity, they do *not necessarily* adhere to physical laws and/or domain-specific constraints. To ensure such an adherence, ASP will be employed as a filtering mechanism that enforces the desired constraints. To that end, the SR output is, first, translated into ASP-compatible facts. Additionally, the presence of specific terms that may violate physical constraints, such as non-physical exponents or the inclusion of essential parameters like the Reynolds number ($\textrm{Re}$) can be identified. Such an ASP encoding is, indicatively, presented subsequently for the equations corresponding to ${\textbf {ID 7}}$ and ${\textbf {ID 9}}$ of Table 3. Note that, as ASP cannot process real numbers, the loss values have been rounded to the nearest integers for compatibility. For a comprehensive discussion of ASP’s syntax and semantics, the interested reader is referred to the Potassco guide^40^.
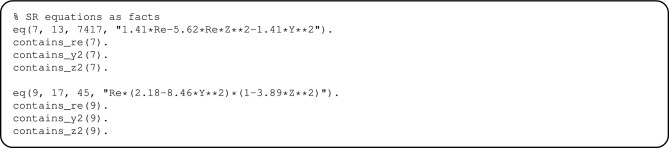


In the above code snippet, the predicate eq/4 represents an SR equation in the form eq(ID, Complexity, Loss, ”Expression”), where the four arguments encapsulate essential information about the equation. The first argument, ID, uniquely identifies the equation within the dataset, allowing for efficient referencing and filtering. The second argument, Complexity, denotes the complexity of the equation. The third argument, Loss, specifies the MSE of the equation when evaluated against the dataset. Finally, the fourth argument, ”Expression”, is a string representation of the symbolic equation itself. An indicative fact that complements an equation is expressed via the predicate contains_re(7), which states that the equation corresponding to ${\textbf {ID 7}}$ includes the Reynolds number ($\textrm{Re}$).

Thereafter, we can impose constraints such as maximum allowable complexity and loss, exclusion of non-physical terms (e.g., $X^3$, $Y^3$, $X^4$), and the requirement that selected models incorporate essential physical parameters like the Reynolds number ($\textrm{Re}$). Such requirements can be represented in an ASP format as follows:
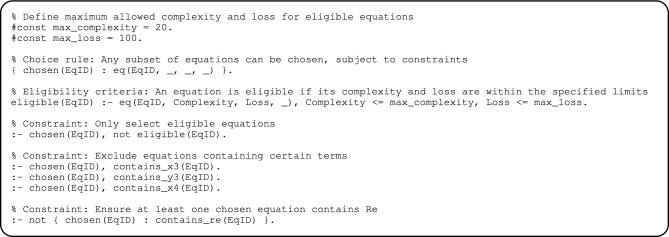


Upon formulating the ASP program, the Clasp ASP solver is employed to process the program, and identify stable models (answer sets) that satisfy all the imposed constraints, evaluating each candidate equation against the eligibility criteria and exclusion constraints. After executing the ASP solver, we derive the following selected/filtered equations in approximately 0.1 seconds — notably, the first equation corresponds to Equation (6) of section Results and discussion.
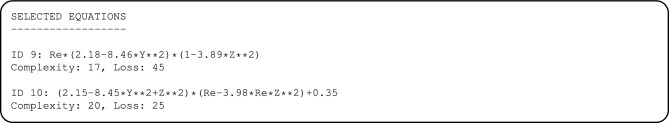


Evidently, the expressions appearing in the list of “SELECTED EQUATIONS” strike an ideal trade-off between accuracy and simplicity (as they are part of the Pareto front of the SR procedure) and, at the same time, they are physically valid (as they adhere to the imposed domain-specific constraints of ASP).

The computation time required by the Clasp ASP solver to generate stable models in our representative example is indeed negligible — approximately 0.1 seconds, which is significantly less than the computation time (around 15 minutes) required by the SR model to derive the equations of the Pareto front (see section Results and discussion). Nevertheless, in more complex fluid-flow problems and geometries, the sheer volume of potential SR equations and the nature of domain-specific constraints can significantly enlarge the search space for stable models and, by extension, increase computational overhead. To mitigate these concerns, one can exploit incremental solving strategies (where constraints are added iteratively), utilize domain-decomposition techniques to tackle sub-problems in parallel, or take advantage of advanced ASP features, such as heuristic-guided optimization, which can substantially narrow the search space^22^. These practices can allow the introduced framework to remain viable in larger, more intricate fluid-mechanics scenarios, while keeping computation times in check.

### More expressive physical constraints

The constraints discussed so far are mostly “syntactic”, but ASP’s expressive language can also encode genuinely physical constraints. In this subsection, we present a representative collection of such constraints.

**No-Slip Boundary Condition** 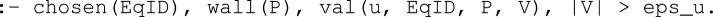


In this constraint, the predicate wall(P) marks each point P that lies on a duct wall (these points are defined in advance). The predicate val(u, EqID, P, V) provides the numerically evaluated value V of the candidate velocity equation EqID at point P. The comparison |V|> eps_u checks whether the magnitude of this value exceeds a small predefined tolerance eps_u, used to represent “effectively zero”. If all these conditions hold simultaneously, the candidate equation violates the no-slip boundary condition. Consequently, ASP eliminates any equation that produces a non-zero wall velocity, thereby ensuring that admissible symbolic expressions satisfy the requirement that the axial velocity vanishes at all wall locations.

**Centerline Symmetry** 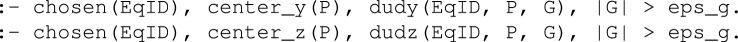


In a rectangular duct, the velocity field is symmetric with respect to the mid-planes $Y = 0$ and $Z = 0$. This symmetry implies that the transverse gradients of the axial velocity must vanish at these locations, that is,

$$\frac{\partial u}{\partial Y} = 0 \quad \text {at} \quad Y = 0 \qquad \text {and} \qquad \frac{\partial u}{\partial Z} = 0 \quad \text {at} \quad Z = 0.$$

Accordingly, in the above constraint, the predicate center_y(P) (resp., center_z(P)) identifies every point P located on the symmetry plane $Y=0$ (resp., $Z=0$). The predicates dudy(EqID, P, G) and dudz(EqID, P, G) represent the evaluated transverse gradients $\partial u/\partial Y$ and $\partial u/\partial Z$ of the candidate velocity equation EqID at point P. The comparison |G|> eps_g checks whether the magnitude of these gradients exceeds a small tolerance eps_g. Whenever this occurs, the constraint is triggered and the candidate equation is eliminated. In this way, the ASP module filters out symbolic expressions that violate the geometric symmetry of the flow.

**Symmetry and Laminar Scaling** 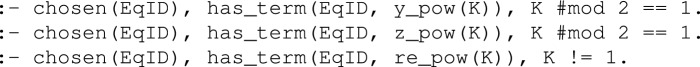


The geometry of the rectangular duct is symmetric with respect to the transverse coordinates $Y$ and $Z$, and the corresponding laminar velocity field inherits this symmetry. As a consequence, physically admissible expressions for the axial velocity must depend only on even powers of $Y$ and $Z$. In addition, in the considered laminar regime, the axial velocity scales linearly with the Reynolds number $\textrm{Re}$, and thus non-linear powers of $\textrm{Re}$ are physically inconsistent. In the ASP rules above, the predicate has_term(EqID, y_pow(K)) (resp., z_pow(K)) indicates that the symbolic expression associated with the equation EqID contains a term proportional to $Y^K$ (resp., $Z^K$). The condition K #mod 2 == 1 (modulo operation) identifies odd exponents, which violate geometric symmetry. Similarly, the predicate has_term(EqID, re_pow(K)) detects terms involving powers of $\textrm{Re}$, and the condition K != 1 filters out non-linear dependencies. Whenever any of these conditions hold, the corresponding constraint is triggered and the candidate equation is rejected. This ensures that the ASP module retains only symbolic expressions that respect the symmetry of the flow and the appropriate laminar scaling with the Reynolds number.

### Final remarks

We conclude our discussion by noting that the proposed approach is sufficiently flexible to recover different analytical forms when applied to datasets reflecting other physical scenarios. For example, in the case of fluid flow in a cylindrical pipe, commonly referred to as Hagen–Poiseuille flow^37^, and using boundary conditions analogous to those adopted for the rectangular-duct case together with a comparable pressure gradient, an SR model developed for this configuration recovered the expected Cartesian form of the axial velocity field, namely the Hagen–Poiseuille profile, with quadratic dependence on the transverse coordinates through $Y^2+Z^2$ and linear parametric scaling with the Reynolds number $\textrm{Re}$. More specifically, the derived expression

$$u^{\textrm{symb}} = 10.18\ \textrm{Re}\,\Big (0.256 - Y^2 - Z^2\Big )$$

is equivalent, in terms of its functional form, to the known analytical parabolic profile $u \propto (C - Y^2 - Z^2)$, where the constant *C* and the multiplicative coefficient depend on the specific flow parameters and determine the overall scale of the velocity field. This additional example further supports the view that, by adjusting the SR search space and tailoring the ASP constraints to the relevant boundary conditions and physical requirements, the framework can be naturally extended to alternative geometries, flow regimes, or governing equations without altering its core methodology.

Finally, we note that the SR/ASP hybrid framework can, to a certain extent, be paralleled with *Physics-Informed Neural Networks* (PINNs)^41,42^. These specialized Artificial Neural Networks embed physical laws directly into their learning process, producing models that are inherently consistent with the underlying physics of the application. Analogously, the SR/ASP hybrid approach capitalizes on domain knowledge to refine and validate the SR equations, thus achieving greater alignment with the physical principles governing a problem.

## Conclusions

Recognizing that understanding flow physics is as crucial as accurate prediction, we presented a Symbolic-Regression (SR) approach for modelling laminar flow in a three-dimensional channel. Using Finite Volume Method (FVM)-generated simulation data and the PySR library, we derived interpretable equations for the velocity and pressure fields without prior assumptions on their functional forms.

The channel geometry featured a rectangular cross-section, with flow characterized by a parabolic velocity profile and linear pressure drop. SR successfully recovered these patterns, as the velocity equation showed quadratic dependence on the vertical (*Y*) and lateral (*Z*) coordinates, while the pressure model captured the expected linear decay along the longitudinal direction (*X*). Both models achieved excellent accuracy, with Normalized Mean Absolute Errors below $0.01\%$ for velocity and $10^{-8}\%$ for pressure, closely matching analytical solutions.

While SR provides accurate, data-driven models, it can overlook domain-specific constraints. To address this, we introduced a hybrid framework that integrates Answer Set Programming (ASP) to enforce physical plausibility and domain rules. This ensures symbolic models remain both statistically robust and physically consistent.

The proposed SR/ASP methodology highlights the potential of combining data-driven and symbolic Artificial Intelligence for fluid dynamics, where interpretability and physical consistency are critical. Future work will extend the SR framework to more complex flows, examine the effect of spatial grid resolution on the recovered symbolic expressions, and explore the use of Chebyshev polynomials to further improve precision, while leveraging Large Language Models to help automate constraint generation for the ASP module.

## Data Availability

The datasets generated during the current study are available from the corresponding author on reasonable request.
